# Supplementary material for: Device-Assessed Physical Activity and Sedentary Behaviors in Canadians with Chronic Disease(s): Findings from the Canadian Health Measures Survey
Source: Sports (Basel). 2019 May 16;7(5):113. doi: 10.3390/sports7050113 (PMC6571578; doi:10.3390/sports7050113)
Supplement: Supplementary file 1 [file sports-07-00113-s001.pdf]

sports

MDPI

Supplementary File

DESCRIPTIVE DATA 2
Table 1 - Characteristics of physical activity and sedentary behavior by disease 2
MULTIVARIATE ANALYSIS 3
Table 2 - Weighted ANCOVA table for CD and MVPA 3
Table 3 - Weighted ANCOVA table for CD and LPA 4
Table 4 - Weighted ANCOVA table for CD and steps 5
Table 5 - Weighted ANCOVA table for CD and SB 6
Table 6 - Weighted ANCOVA table for heart disease and MVPA 7
Table 7 - Weighted ANCOVA table for heart disease and LPA 8
Table 8 - Weighted ANCOVA table for heart disease and steps 9
Table 9 - Weighted ANCOVA table for heart disease and SB 10
Table 10 - Weighted ANCOVA table for cancer and MVPA 11
Table 11 - Weighted ANCOVA table for cancer and LPA 12
Table 12 - Weighted ANCOVA table for cancer and steps 13
Table 13 - Weighted ANCOVA table for cancer and SB 14
Table 14 - Weighted ANCOVA table for diabetes and MVPA 15
Table 15 - Weighted ANCOVA table for diabetes and LPA 16
Table 16 - Weighted ANCOVA table for diabetes and steps 17
Table 17 - Weighted ANCOVA table for diabetes and SB 18

DESCRIPTIVE DATA

Table S1. - Characteristics of physical activity and sedentary behavior by disease.

	MVPA (min/day)				LPA (min/day)				Steps (steps/day)				SB (min/day)			
	M	Se	Md	IQR	M	Se	Md	IQR	M	Se	Md	IQR	M	Se	Md	IQR
**0 disease**	19.8	0.7	14.4	21.7	218.1	2.6	208.1	102.7	7817	104.6	7383	4604	555.3	2.5	561.3	123
**1 disease**	15.6	1	8.6	18.1	205.9	4.2	195.8	106.8	7291	177.8	6645	4876	564.2	4.2	563.3	118.8
**2 disease**	9.7	0.9	4.6	12	171.4	7	156.7	102.3	5779	303.5	5186	4133	579.7	6.4	578.6	126.8
**3 or + diseases**	6.4	1	3	6.8	155.4	9.6	153.1	79.4	5483	666.9	4926	3616	606.8	16.1	602.7	99.2
**HD**	10.2	0.7	7.3	12.6	174.1	5.3	167.5	98.6	5991	204.1	5277	4241	463.1	15.7	541.2	381.9
**Cancer**	16.8	2.3	9.1	20	188.9	5	185.6	100.7	6832	328	6235	4725	477.5	11.6	538.3	291.6
**Diabetes**	12.3	2	6.6	15.4	174.1	11.1	156.2	87.6	6116	483.2	5397	3944	481.4	20.5	543.1	283.2

Note: HD is Heart disease, COPD is Chronic obstructive pulmonary disease, M is Mean, Se is Standard error, Md is Median, IQR is Interquartile range.

MULTIVARIATE ANALYSIS

Table S2. - Weighted ANCOVA table for CD and MVPA.

(controlling for age, sex, body mass index, time of wearing the accelerometer, marital status, self-perceived health, self-perceived mental health, working status, season, smoking status (blood cotinine levels), education level and total household income).

	Estimate	SE	95% CI	t	*p*
1 CD	-0.53	0.05	-0.16 – 0.05	-1	0.32
2 CD	-0.33	0.07	-0.47 – -0.2	-4.83	0.0002E-02
3+ CD	-0.83	0.17	-1.16 – -0.51	-5.02	0.0007E-03
Age	-0.01	0.02	-0.01 – -0.006	-5.73	0.0002E-04
BMI	-0.03	0.005	-0.04 – -0.03	-7.36	0.0008E-09
Sex (women)	-0.28	0.04	-0.36 – -0.2	-7.18	0.0003E-08
Accelerometer wearing	0.09	0.01	0.07 – 0.12	8.13	0.0004E-11
Marital status (couple)	-0.19	0.06	-0.3 – -0.08	-3.28	0.001
Self-rated health	-0.43	0.08	-0.59 – -0.27	-5.26	0.0002E-03
Self-rated mental health	0.2	0.12	-0.03 – 0.44	1.68	0.09
Worked last year					
Study/retired	-0.2	0.13	-0.46 – 0.06	-1.48	0.14
Working	0.09	0.07	-0.05 – 0.22	1.29	0.2
Seasons					
Spring	-0.005	0.08	-0.16 – 0.15	-0.07	0.95
Summer	-0.06	0.06	-0.17 – 0.15	-1.06	0.29
Winter	-0.12	0.1	-0.32 – 0.09	-1.13	0.26
Levels of cotinine	-0.0002	0.0004E-01	-0.0003 – -0.0001	-5.51	0.0006E-04
Education					
High school	0.13	0.12	-0.1 – 0.37	1.11	0.27
Work school	0.07	0.1	-0.13 – 0.27	0.68	0.49
College	0.12	0.11	-0.1 – 0.34	1.07	0.28
University < Bachelor	0.21	0.1	0.01 – 0.4	2.09	0.04
Bachelor	0.33	0.13	0.08 – 0.57	2.58	0.01
University > Bachelor	0.32	0.11	0.09 – 0.54	2.78	0.006
Missing	0.08	0.1	-0.12 – 0.28	0.8	0.42
Household income					
$15k-$19,99k	-0.14	0.19	-0.52 – 0.24	-0.72	0.47
$20k-$29,99k	-0.25	0.15	-0.54 – 0.05	-1.66	0.1
$30k-$39,99k	-0.21	0.13	-0.47 – 0.05	-1.59	0.11
$40k-$49,99k	-0.17	0.15	-0.46 – 0.13	-1.11	0.27
$50k-$59,99k	-0.21	0.16	-0.52 – 0.1	-1.34	0.18
$60k-$79,99k	-0.09	0.14	-0.37 – 0.19	-0.61	0.54
$80k-$99,99k	-0.006	0.15	-0.29 – 0.28	-0.04	0.97
≥ $100k	0.03	0.14	-0.25 – 0.3	0.18	0.86

Table S3. - Weighted ANCOVA table for CD and LPA.

(controlling for age, sex, body mass index, time of wearing the accelerometer, marital status, self-perceived health, self-perceived mental health, working status, season, smoking status (blood cotinine levels), education level and total household income).

	**Estimate**	**SE**	**95% CI**	**t**	***p***
1 CD	-1.18	3.22	-7.5 – 5.14	-0.37	0.71
2 CD	-13.91	4.94	-23.59 – -4.23	-2.82	0.005
3+ CD	-30.54	7.37	-44.99 – -16.08	-4.14	0.0004E-01
Age	-0.77	0.15	-1.05 – -0.48	-5.25	0.0002E-03
BMI	-0.26	0.29	-0.82 – 0.31	-0.9	0.37
Sex (women)	-7.42	3.13	-13.57 – -1.28	-2.37	0.02
Accelerometer wearing	26.29	1.1	24.13 – 28.44	23.92	0.0002E-12
Marital status (couple)	6.75	3.84	-0.77 – 14.27	1.76	0.08
Self-rated health	-15.41	5.11	-25.42 – -5.4	-3.02	0.003
Self-rated mental health	-14.1	9	-31.74 – 3.54	-1.57	0.12
Worked last year					
Study/retired	-6.6	5.69	-17.76 – 4.57	-1.16	0.25
Working	11.77	3.66	4.6 – 18.93	3.22	0.001
Seasons					
Spring	3.5	3.73	-3.81 – 10.81	0.94	0.35
Summer	3.78	5.82	-7.64 – 15.19	0.65	0.52
Winter	-14.86	4.84	-24.36 – -5.37	-3.07	0.002
Levels of cotinine	-0.005	0.003	-0.01 – 0.0002	-1.9	0.06
Education					
High school	20.27	11	-1.29 – 41.84	1.84	0.07
Work school	3.28	10.03	-16.37 – 22.93	0.33	0.74
College	-0.55	8.99	-18.16 – 17.07	-0.06	0.95
University < Bachelor	-3.95	8.58	-20.77 – 12.86	-0.46	0.65
Bachelor	-13.49	9.27	-31.67 – 4.69	-1.46	0.15
University > Bachelor	-19.53	10.8	-40.71 – 1.64	-1.81	0.07
Missing	18.89	7.89	3.43 – 34.35	2.39	0.02
Household income					
$15k-$19,99k	-0.19	12.76	-25.19 – 24.81	-0.02	0.99
$20k-$29,99k	2.84	9.99	-16.75 – 22.43	0.28	0.78
$30k-$39,99k	5.55	8.87	-11.84 – 22.94	0.63	0.53
$40k-$49,99k	9.41	11.88	-13.86 – 32.69	0.79	0.43
$50k-$59,99k	11.9	9.15	-6.04 – 29.85	1.3	0.19
$60k-$79,99k	12.19	8.12	-3.72 – 28.11	1.5	0.13
$80k-$99,99k	15.74	8.52	-0.95 – 32.44	1.85	0.07
≥ $100k	1.74	7.59	-13.13 – 16.61	0.23	0.82

Table S4. - Weighted ANCOVA table for CD and steps.

(controlling for age, sex, body mass index, time of wearing the accelerometer, marital status, self-perceived health, self-perceived mental health, working status, season, smoking status (blood cotinine levels), education level and total household income).

	**Estimate**	**SE**	**95% CI**	**t**	***p***
1 CD	-21.26	153.16	-321.45 – 278.93	-0.14	0.89
2 CD	-784.03	205.17	-1186.16 – -381.91	-3.82	0.0002
3+ CD	-1547.41	483.6	-2495.25 – -599.56	-3.2	0.001
Age	-16.9	6.48	-29.61 – -4.2	-2.61	0.009
BMI	-84.91	14.23	-112.8 – -57.03	-5.97	0.0005E-05
Sex (women)	-912.25	146.32	-1199.04 – -625.46	-6.23	0.0002E-12
Accelerometer wearing	875.98	52.8	772.49 – 979.47	16.59	0.36
Marital status (couple)	-181.09	197.02	-567.24 – 205.06	-0.92	0.0003E-02
Self-rated health	-988.04	210.09	-1399.82 – -576.27	-4.7	0.13
Self-rated mental health	-479.32	315.32	-1097.33 – 138.7	-1.52	0.8
Worked last year					
Study/retired	-73.89	295.96	-653.96 – 506.18	-0.25	0.8
Working	471.57	212.19	55.7 – 887.5	2.22	0.03
Seasons					
Spring	95.61	225.81	-346.97 – 538.19	0.42	0.67
Summer	90.18	214.11	-329.47 – 509.83	0.42	0.67
Winter	-825	226.66	-1269.25 – -380.76	-3.64	0.0003
Levels of cotinine	-0.49	0.14	-0.77 – -0.21	-3.46	0.0006
Education					
High school	784.66	380.32	39.24 – 1530.07	2.06	0.04
Work school	436.28	379.16	-306.86 – 1179.43	1.15	0.25
College	93.9	366.66	-624.74 – 812.54	0.26	0.8
University < Bachelor	660.93	327.48	19.08 – 1302.79	2.02	0.04
Bachelor	598.33	391.05	-168.11 – 1364.77	1.53	0.13
University > Bachelor	753.8	451.01	-130.16 – 1637.77	1.67	0.1
Missing	956.78	355.31	260.34 –1653.17	2.69	0.007
Household income					
$15k-$19,99k	-535.54	491.86	-1499.56 – 428.48	-1.09	0.28
$20k-$29,99k	-347.09	470.2	-1268.67 – 574.49	-0.74	0.46
$30k-$39,99k	-301.98	412.37	-1110.22 – 506.26	-0.73	0.46
$40k-$49,99k	-214.67	516.33	-1226.66 – 797.32	-0.42	0.68
$50k-$59,99k	123.63	417.04	-693.74 – 941.01	0.3	0.77
$60k-$79,99k	-67.98	396.79	-845.68 – 709.72	-0.17	0.86
$80k-$99,99k	345.39	418	-473.87 – 1164.65	0.83	0.41
≥ $100k	224.72	366.55	-493.71 – 943.15	0.61	0.54

Table S5. - Weighted ANCOVA table for CD and SB.

(controlling for age, sex, body mass index, time of wearing the accelerometer, marital status, self-perceived health, self-perceived mental health, working status, season, smoking status (blood cotinine levels), education level and total household income).

	**Estimate**	**SE**	**95% CI**	**t**	***p***
1 CD	2.14	4.72	-7.11 – 11.39	0.45	0.65
2 CD	17.54	6.6	4.61 – 30.47	2.66	0.008
3+ CD	39.97	12.99	14.51 – 65.43	3.08	0.002
Age	1.8	0.19	1.44 – 2.16	9.71	0.0002e-12
BMI	0.31	0.34	-0.35 – 0.96	0.91	0.36
Sex (women)	16.36	4.4	7.74 – 24.98	3.72	0.0002
Accelerometer wearing	34.77	1.22	32.38 – 37.16	28.5	0.0002E-12
Marital status (couple)	-0.94	3.42	-7.65 – 5.77	-0.27	0.78
Self-rated health	18.75	4.83	9.29 – 28.22	3.88	0.0001
Self-rated mental health	8.22	9.37	-10.13 – 26.58	0.88	0.38
Worked last year					
Study/retired	-7.45	5.97	-19.16 – 4.25	-1.25	0.21
Working	-21.79	5.37	-32.31 – -11.27	-4.06	0.0006E-01
Seasons					
Spring	-1.51	4.88	-11.08 – 8.05	-0.31	0.76
Summer	-12.59	6.53	-25.4 – 0.21	-1.93	0.05
Winter	14.34	5.98	2.63 – 26.05	2.4	0.02
Levels of cotinine	-0.001	0.004	-0.009 – 0.007	-0.3	0.76
Education					
High school	-6.98	10.74	-28.02 – 14.06	-0.65	0.52
Work school	14.45	10.77	-6.65 – 35.55	1.34	0.18
College	13.51	11.55	-9.14 – 36.14	1.17	0.24
University < Bachelor	23.76	9.82	4.52 – 43.01	2.42	0.02
Bachelor	22.12	9.79	2.93 – 41.3	2.26	0.02
University > Bachelor	18.38	14.99	-11.01 – 47.76	1.23	0.22
Missing	-5.7	8.21	-21.8 – 10.39	-0.7	0.49
Household income					
$15k-$19,99k	9.29	14.23	-18.61 – 37.19	0.65	0.51
$20k-$29,99k	8.78	13.68	-18.02 – 35.59	0.64	0.52
$30k-$39,99k	2.52	13.37	-23.68 – 28.73	0.19	0.85
$40k-$49,99k	-1.62	13.56	-28.19 – 24.95	-0.12	0.9
$50k-$59,99k	5.25	12.78	-19.8 – 30.30	0.41	0.68
$60k-$79,99k	-5.9	12.71	-30.81 – 19.01	-0.46	0.64
$80k-$99,99k	-1.94	12.96	-27.34 – 23.47	-0.15	0.88
≥ $100k	6.85	12	-16.68 – 30.37	0.57	0.57

Table S6. - Weighted ANCOVA table for heart disease and MVPA.

(controlling for age, sex, body mass index, time of wearing the accelerometer, marital status, self-perceived health, self-perceived mental health, working status, season, smoking status (blood cotinine levels), education level and total household income).

	**Estimate**	**SE**	**95% CI**	**t**	***p***
Heart disease	-0.36	0.08	-0.51 – -0.21	-4.71	0.0003E-02
Age	-0.01	0.002	-0.01 – -0.007	-5.96	0.0005E-05
BMI	-0.03	0.005	-0.04 – -0.03	-7.62	0.0001E-09
Sex (women)	-0.28	0.04	-0.35 – -0.2	-7.33	0.0001E-08
Accelerometer wearing	0.1	0.01	0.08 – 0.12	8.47	0.0003E-12
Marital status (couple)	-0.18	0.06	-0.29 – -0.06	-3	0.003
Self-rated health	-0.4	0.07	-0.54 – -0.26	-5.58	0.0004E-04
Self-rated mental health	0.07	0.08	-0.09 – 0.23	0.86	0.39
Worked last year					
Study/retired	-0.22	0.13	-0.48 – 0.05	-1.61	0.11
Working	0.09	0.07	-0.04 – 0.22	1.34	0.18
Seasons					
Spring	-0.003	0.08	-0.15 – 0.15	-0.03	0.97
Summer	-0.05	0.06	-0.16 – 0.06	-0.92	0.36
Winter	-0.13	0.1	-0.33 – 0.07	-1.25	0.21
Levels of cotinine	-0.0002	0.0003E-01	-0.0003 – -0.0001	-6.11	0.0002E-05
Education					
High school	0.09	0.11	-0.13 – 0.31	0.81	0.42
Work school	0.08	0.1	-0.12 – 0.28	0.79	0.43
College	0.12	0.11	-0.09 – 0.34	1.14	0.25
University < Bachelor	0.21	0.1	0.02 – 0.4	2.16	0.03
Bachelor	0.32	0.12	0.08 – 0.57	2.62	0.009
University > Bachelor	0.32	0.11	0.1 – 0.54	2.89	0.004
Missing	0.09	0.1	-0.11 – 0.28	0.88	0.38
Household income					
$15k-$19,99k	-0.18	0.19	-0.56 – 0.2	-0.94	0.35
$20k-$29,99k	-0.26	0.14	-0.55 – 0.02	-1.83	0.07
$30k-$39,99k	-0.28	0.13	-0.54 – -0.02	-2.14	0.03
$40k-$49,99k	-0.19	0.14	-0.47 – 0.08	-1.37	0.17
$50k-$59,99k	-0.24	0.15	-0.54 – 0.06	-1.56	0.12
$60k-$79,99k	-0.11	0.13	-0.37 – 0.16	-0.79	0.43
$80k-$99,99k	-0.03	0.14	-0.31 – 0.24	-0.25	0.81
≥ $100k	-0.001	0.13	-0.26 – 0.26	-0.007	0.99

Table S7. - Weighted ANCOVA table for heart disease and LPA.

(controlling for age, sex, body mass index, time of wearing the accelerometer, marital status, self-perceived health, self-perceived mental health, working status, season, smoking status (blood cotinine levels), education level and total household income).

	**Estimate**	**SE**	**95% CI**	**t**	***p***
Heart disease	-19.41	4.87	-28.96 – -9.86	-3.98	0.0008E-01
Age	-0.79	0.14	-1.06 – -0.52	-5.78	0.0001E-04
BMI	-0.2	0.28	-0.76 – 0.36	-0.71	0.48
Sex (women)	-5.98	2.78	-11.4 – -0.53	-2.15	0.03
Accelerometer wearing	26	1.02	24 – 27.99	25.59	0.0002E-12
Marital status (couple)	6.2	3.42	-0.5 – 12.92	1.81	0.07
Self-rated health	-15.28	4.46	-24.02 – -6.54	-3.43	0.0007
Self-rated mental health	-5.2	4.8	-14.61 – 4.22	-1.08	0.28
Worked last year					
Study/retired	-4.07	5.08	-14.04 – 5.89	-0.8	0.42
Working	14.41	3.4	7.75 – 21.07	4.24	0.0003E-01
Seasons					
Spring	3.17	3.84	-4.35 – 10.69	0.83	0.41
Summer	4.25	5.46	-6.44 – 14.95	0.78	0.44
Winter	-13.73	4.74	-23.02 – -4.44	-2.9	0.004
Levels of cotinine	-0.004	0.003	-0.009 – 0.002	-1.41	0.16
Education					
High school	19.91	10.36	-0.39 – 40.21	1.92	0.06
Work school	0.13	10.33	-20.12 – 20.38	0.01	0.99
College	-5.43	9.05	-23.18 – 12.31	-0.6	0.55
University < Bachelor	-7.83	8.82	-25.17 – 9.45	-0.89	0.37
Bachelor	-15.58	9.65	-34.5 – 3.34	-1.61	0.11
University > Bachelor	-23.78	10.77	-44.9 – -2.67	-2.21	0.03
Missing	15.32	8.23	-0.81 – 31.45	1.86	0.06
Household income					
$15k-$19,99k	-0.61	11.87	-23.88 – 22.67	-0.05	0.96
$20k-$29,99k	1.96	9.5	-16.66 – 20.58	0.21	0.84
$30k-$39,99k	6.84	8.31	-9.45 – 23.13	0.82	0.41
$40k-$49,99k	13.68	9.37	-4.68 – 32.03	1.46	0.14
$50k-$59,99k	14.76	7.98	-0.89 – 30.41	1.85	0.07
$60k-$79,99k	13.66	7.5	-1.04 – 28.36	1.82	0.07
$80k-$99,99k	16.66	8.16	0.68 – 32.65	2.04	0.04
≥ $100k	3.97	7.31	-10.36 – 18.3	0.54	0.59

Table S8. - Weighted ANCOVA table for heart disease and steps.

(controlling for age, sex, body mass index, time of wearing the accelerometer, marital status, self-perceived health, self-perceived mental health, working status, season, smoking status (blood cotinine levels), education level and total household income).

	**Estimate**	**SE**	**95% CI**	**t**	***p***
Heart disease	-947.11	270.47	-1477.23 – -417	-3.5	0.0005
Age	-17.61	6.6	-30.54 – -4.68	-2.67	0.008
BMI	-85.28	14.08	-112.88 – -57.69	-6.06	0.0003E-05
Sex (women)	-916.01	141.97	-1194.3 – -637.74	-6.45	0.0003E-06
Accelerometer wearing	871.52	52.18	769.24 – 973.79	16.7	0.0002E-12
Marital status (couple)	-180.54	192.21	-557.26 – 196.18	-0.94	0.35
Self-rated health	-970.11	207.63	-1377.06 – -563.17	-4.67	0.0004E-02
Self-rated mental health	-482.5	301.53	-1073.48 – 108.47	-1.6	0.11
Worked last year					
Study/retired	-24.01	292.8	-597.88 – 549.86	-0.08	0.93
Working	521.22	211.13	107.41 – 935.03	2.47	0.01
Seasons					
Spring	122.08	214.1	-297.54 – 541.71	0.57	0.58
Summer	129.24	204.63	-271.84 – 530.31	0.63	0.53
Winter	-798.2	217.82	-1225.17 – -371.28	-3.67	0.0003
Levels of cotinine	-0.004	0.14	-0.78 – -0.23	-3.63	0.0003
Education					
High school	762.38	373.3	30.72 – 1494.04	2.04	0.04
Work school	461.01	377.83	-279.53 – 1201.55	1.22	0.22
College	75.5	353.39	-617.13 – 768.12	0.21	0.83
University < Bachelor	651.2	322.9	18.33 – 1284.07	2.02	0.04
Bachelor	560.27	387.24	-198.71 – 1319.26	1.45	0.15
University > Bachelor	727.14	422.75	-140.64 – 1594.91	1.64	0.1
Missing	952.25	351.83	262.68 – 1641.82	2.71	0.007
Household income					
$15k-$19,99k	-651.77	465.21	-1563.55 – 260.02	-1.4	0.16
$20k-$29,99k	-429.95	442.42	-1297.08 – 437.18	-0.97	0.33
$30k-$39,99k	-318.8	390.4	-1083.96 – 446.36	-0.82	0.41
$40k-$49,99k	-299.47	477.17	-1234.71 – 635.78	-0.63	0.53
$50k-$59,99k	49.9	385.85	-706.34 – 806.14	0.13	0.9
$60k-$79,99k	-84.38	359.44	-788.87 – 620.11	-0.24	0.81
$80k-$99,99k	278.61	389.81	-485.42 – 1042.63	0.72	0.48
≥ $100k	179.54	337.9	-482.73 – 841.81	0.53	0.6

Table S9. - Weighted ANCOVA table for heart disease and SB.

(controlling for age, sex, body mass index, time of wearing the accelerometer, marital status, self-perceived health, self-perceived mental health, working status, season, smoking status (blood cotinine levels), education level and total household income).

	**Estimate**	**SE**	**95% CI**	**t**	***p***
Heart disease	21.95	7.66	6.93 – 36.96	2.87	0.004
Age	1.83	0.18	1.48 – 2.18	10.26	0.0002E-12
BMI	0.33	0.33	-0.32 – 0.97	1	0.32
Sex (women)	16.49	4.14	8.38 – 24.61	3.98	0.0008E-01
Accelerometer wearing	34.83	1.19	32.5 – 37.15	29.31	0.0002E-12
Marital status (couple)	-0.64	3.37	-7.26 – 5.97	-0.19	0.85
Self-rated health	17.59	4.81	8.17 – 27.02	3.66	0.0003
Self-rated mental health	7.8	8.94	-9.73 – 25.32	0.87	0.38
Worked last year					
Study/retired	-8.2	5.95	-19.87 – 3.46	-1.38	0.17
Working	-22.57	5.12	-32.6 – -12.54	-4.41	0.0001E-01
Seasons					
Spring	-2.17	4.86	-11.69 – 7.35	-0.45	0.66
Summer	-13.3	6.37	-25.78 – -0.82	-2.09	0.04
Winter	13.67	5.94	2.04 – 25.31	2.3	0.02
Levels of cotinine	-0.0005	0.004	-0.008 – 0.007	-0.13	0.9
Education					
High school	-6.46	10.41	-26.87 – 13.95	-0.62	0.54
Work school	13.83	10.53	-6.83 – 34.45	1.31	0.19
College	14.16	11.53	-8.43 – 36.75	1.23	0.22
University < Bachelor	24.01	9.6	5.2 – 42.83	2.5	0.01
Bachelor	22.88	9.59	4.09 – 41.66	2.39	0.02
University > Bachelor	18.96	14.86	-0.1 – 48.09	1.28	0.2
Missing	-6.1	8	-21.78 – 9.59	-0.76	0.45
Household income					
$15k-$19,99k	10.2	13.37	-16.01 – 36.41	0.76	0.45
$20k-$29,99k	9.46	12.7	-15.43 – 34.35	0.75	0.46
$30k-$39,99k	1.81	12.62	-22.93 – 26.54	0.14	0.89
$40k-$49,99k	-1.65	12.47	-26.09 – 22.79	-0.13	0.89
$50k-$59,99k	5.27	11.74	-17.74 – 28.28	0.45	0.65
$60k-$79,99k	-6.63	11.92	-29.99 – 16.72	-0.56	0.58
$80k-$99,99k	-1.95	12.5	-26.45 – 22.55	-0.16	0.88
≥ $100k	6.16	11	-15.39 – 27.72	0.56	0.58

Table S10. - Weighted ANCOVA table for cancer and MVPA.

(controlling for age, sex, body mass index, time of wearing the accelerometer, marital status, self-perceived health, self-perceived mental health, working status, season, smoking status (blood cotinine levels), education level and total household income).

	**Estimate**	**SE**	**95% CI**	**t**	***p***
Cancer	-0.13	0.06	-0.25 – -0.01	-2.13	0.03
Age	-0.01	0.002	-0.01 – -0.008	-6.61	0.0001E-06
BMI	-0.03	0.005	-0.04 – -0.03	-7.54	0.0002E-09
Sex (women)	-0.27	0.04	-0.34 – -0.2	-7.15	0.0003E-08
Accelerometer wearing	0.09	0.01	0.07 – 0.12	8.04	0.0008E-11
Marital status (couple)	-0.18	0.06	-0.29 – -0.06	-3.06	0.002
Self-rated health	-0.45	0.08	-0.61 – -0.29	-5.58	0.0004E-04
Self-rated mental health	0.19	0.12	-0.04 – 0.42	1.6	0.11
Worked last year					
Study/retired	-0.21	0.13	-0.47 – 0.05	-1.61	0.11
Working	0.1	0.07	-0.03 – 0.22	1.44	0.15
Seasons					
Spring	-0.01	0.08	-0.17 – 0.14	-0.18	0.86
Summer	-0.05	0.06	-0.16 – 0.05	-0.98	0.33
Winter	-0.12	0.1	-0.32 – 0.09	-1.14	0.26
Levels of cotinine	-0.0002	0.0003E-01	-0.0003 – -0.0001	-5.85	0.0009E-05
Education					
High school	0.12	0.12	-0.11 – 0.31	1.03	0.3
Work school	0.07	0.1	-0.13 – 0.28	0.68	0.5
College	0.12	0.11	-0.1 – 0.34	1.06	0.29
University < Bachelor	0.2	0.1	0.006 – 0.4	2.02	0.04
Bachelor	0.32	0.13	0.07 – 0.57	2.55	0.01
University > Bachelor	0.32	0.11	0.1 – 0.54	2.83	0.005
Missing	0.08	0.1	-0.12 – 0.28	0.78	0.44
Household income					
\$15k-\$19,99k	-0.16	0.19	-0.53 – 0.21	-0.84	0.4
\$20k-\$29,99k	-0.25	0.14	-0.53 – 0.03	-1.73	0.09
\$30k-\$39,99k	-0.22	0.12	-0.46 – -0.02	-1.79	0.07
\$40k-\$49,99k	-0.18	0.14	-0.45 – 0.1	-1.28	0.2
\$50k-\$59,99k	-0.23	0.15	-0.53 – 0.07	-1.49	0.14
\$60k-\$79,99k	-0.09	0.13	-0.36 – 0.17	-0.67	0.5
\$80k-\$99,99k	-0.01	0.14	-0.28 – 0.26	-0.07	0.94
≥ \$100k	0.02	0.13	-0.24 – 0.28	0.15	0.88

Table S11. - Weighted ANCOVA table for cancer and LPA.

(controlling for age, sex, body mass index, time of wearing the accelerometer, marital status, self-perceived health, self-perceived mental health, working status, season, smoking status (blood cotinine levels), education level and total household income).

	Estimate	SE	95% CI	t	*p*
Cancer	-5.25	3.74	-12.58 – 2.07	-1.41	0.16
Age	-0.83	0.14	-1.09 – -0.56	-6.06	0.0003E-05
BMI	-0.2	0.28	-0.76 – 0.35	-0.71	0.48
Sex (women)	-5.17	2.76	-10.58 – 0.24	-1.87	0.06
Accelerometer wearing	26	1.02	24 – 28	25.62	0.0002E-12
Marital status (couple)	6.56	3.38	-0.06 – 13.19	1.94	0.05
Self-rated health	-16.63	4.4	-25.26 – -8	-3.78	0.0002
Self-rated mental health	-4.22	4.85	-13.74 – 5.29	-0.87	0.38
Worked last year					
Study/retired	-5.14	5.21	-15.36 – 5.07	-0.99	0.32
Working	14.92	3.47	8.12 – 21.73	4.3	0.0002E-01
Seasons					
Spring	2.56	3.88	-5.04 – 10.16	0.66	0.51
Summer	4.09	5.47	-6.63 – 14.82	0.75	0.45
Winter	-13.71	4.78	-23.09 – -4.33	-2.87	0.004
Levels of cotinine	-0.004	0.003	-0.009 – 0.002	-1.36	0.17
Education					
High school	19.36	10.34	-0.9 – 39.63	1.87	0.06
Work school	-0.46	10.43	-20.9 – 19.98	-0.04	0.96
College	-5.43	9.17	-23.39 – 12.54	-0.59	0.55
University < Bachelor	-8.24	8.91	-25.7 – 9.21	-0.93	0.36
Bachelor	-15.5	9.71	-34.54 – 3.53	-1.6	0.11
University > Bachelor	-23.34	10.96	-44.82 – -1.86	-2.13	0.03
Missing	14.91	8.26	-1.27 – 31.09	1.81	0.07
Household income					
$15k-$19,99k	0.81	12.21	-23.12 – 24.75	0.07	0.95
$20k-$29,99k	2.78	9.61	-16.06 – 21.62	0.29	0.77
$30k-$39,99k	7.29	8.57	-9.5 – 24.09	0.85	0.4
$40k-$49,99k	14.19	9.55	-4.54 – 32.92	1.49	0.14
$50k-$59,99k	15.17	8.16	-0.83 – 31.17	1.86	0.06
$60k-$79,99k	13.86	7.75	-1.33 – 29.04	1.79	0.07
$80k-$99,99k	17.44	8.43	0.9 – 33.97	2.07	0.04
≥ $100k	4.37	7.52	-10.36 – 19.1	0.58	0.56

Table S12. - Weighted ANCOVA table for cancer and steps.

(controlling for age, sex, body mass index, time of wearing the accelerometer, marital status, self-perceived health, self-perceived mental health, working status, season, smoking status (blood cotinine levels), education level and total household income).

	Estimate	SE	95% CI	t	*p*
Cancer	-207.26	184.35	-568.58 – 154.06	-1.12	0.26
Age	-19.41	6.48	-32.11 – -6.72	-3	0.003
BMI	-85.97	14.08	-113.57 – -58.36	-6.1	0.0002E-05
Sex (women)	-875.69	139.48	-1149.07 – -602.3	-6.28	0.0008E-06
Accelerometer wearing	872.42	52.27	769.97 – 9734.86	16.69	0.0002E-12
Marital status (couple)	-154	193.01	-532.29 – 224.29	-0.8	0.43
Self-rated health	-1027.02	203.41	-1425.69 – -628.35	-5.05	0.0006E-03
Self-rated mental health	-414.42	308.26	-1018.59 – 189.75	-1.34	0.18
Worked last year					
Study/retired	-75.9	296.62	-657.27 – 505.47	-0.26	0.8
Working	550.36	205.43	147.73 – 952.99	2.68	0.008
Seasons					
Spring	89.74	221.57	-344.53 – 524.02	0.41	0.69
Summer	123.58	202.83	-273.97 – 521.13	0.61	0.54
Winter	-800.79	218.87	-1229.77 – -371.8	-3.65	0.0003
Levels of cotinine	-0.5	0.14	-0.78 – -0.22	-3.53	0.0005
Education					
High school	730.21	371.3	1.82 – 1494.04	1.97	0.05
Work school	423.71	377.83	-316.19 – 1201.55	1.12	0.26
College	79.36	353.39	-620.65 – 768.12	0.22	0.82
University < Bachelor	629.56	322.9	-4.54 – 1284.07	1.95	0.05
Bachelor	560.71	387.24	-199.68 – 1319.26	1.45	0.15
University > Bachelor	743.94	422.75	-134.14 – 1594.91	1.66	0.1
Missing	931.22	351.83	248.43 – 1641.82	2.67	0.01
Household income					
$15k-$19,99k	-545	475.24	-1476.45 – 386.46	-1.15	0.25
$20k-$29,99k	-381.03	444.29	-1251.82 – 489.77	-0.86	0.39
$30k-$39,99k	-291.52	400.62	-1076.71 – 493.67	-0.73	0.47
$40k-$49,99k	-278.74	484.15	-1227.66 – 670.18	-0.58	0.57
$50k-$59,99k	61.17	395.76	-714.52 – 836.85	0.16	0.88
$60k-$79,99k	-71.1	373.24	-802.64 – 660.45	-0.19	0.85
$80k-$99,99k	318.53	399.37	-464.22 – 1101.28	0.8	0.43
≥ $100k	202.37	344.7	-473.22 – 877.96	0.59	0.56

Table S13. - Weighted ANCOVA table for cancer and SB.

(controlling for age, sex, body mass index, time of wearing the accelerometer, marital status, self-perceived health, self-perceived mental health, working status, season, smoking status (blood cotinine levels), education level and total household income).

	**Estimate**	**SE**	**95% CI**	**t**	***p***
Cancer	8.69	4.09	0.67 – 16.7	2.12	0.03
Age	1.85	0.17	1.52 – 2.18	10.87	0.0002E-12
BMI	0.33	0.33	-0.32 – 0.98	0.99	0.32
Sex (women)	15.33	4.23	7.04 – 23.62	3.62	0.0003
Accelerometer wearing	34.79	1.18	32.48 – 37.09	29.58	0.0002E-12
Marital status (couple)	-0.11	3.39	-7.77 – 5.53	-0.33	0.74
Self-rated health	18.95	4.8	9.56 – 28.35	3.95	0.0009E-01
Self-rated mental health	5.99	9.09	-11.84 – 23.81	0.66	0.51
Worked last year					
Study/retired	-7.39	6.16	-19.46 – 4.68	-1.2	0.23
Working	-22.94	5.3	-33.33 – -12.54	-4.33	0.0002E-01
Seasons					
Spring	-1.79	4.79	-11.18 – 7.6	-0.37	0.71
Summer	-13.14	6.27	-25.44 – -0.85	-2.1	0.04
Winter	13.73	5.86	2.25 – 25.22	2.34	0.02
Levels of cotinine	-0.0007	0.004	-0.008 – 0.007	-0.19	0.85
Education					
High school	-5.68	10.5	-26.25 – 14.89	-0.54	0.59
Work school	14.15	10.67	-6.77 – 35.06	1.33	0.19
College	14.37	11.75	-8.65 – 37.39	1.22	0.22
University < Bachelor	24.43	9.77	5.29 – 43.57	0.01	0.01
Bachelor	22.75	9.8	3.55 – 41.95	0.02	0.02
University > Bachelor	18.34	15.08	-11.21 – 47.89	0.22	0.22
Missing	-5.51	8.08	-21.35 – 10.32	0.5	0.5
Household income					
$15k-$19,99k	8.72	13.33	-17.4 – 34.85	0.51	0.51
$20k-$29,99k	8.64	13.03	-16.89 – 34.18	0.51	0.51
$30k-$39,99k	1.16	12.88	-24.08 – 26.4	0.93	0.93
$40k-$49,99k	-2.27	12.76	-27.29 – 22.75	0.86	0.86
$50k-$59,99k	4.72	12.03	-18.85 – 28.29	0.69	0.69
$60k-$79,99k	-7.04	12.15	-30.86 – 16.77	0.56	0.56
$80k-$99,99k	-3.68	12.68	-28.53 – 21.18	0.77	0.77
≥ $100k	5.49	12.29	-16.63 – 27.61	0.63	0.63

Table S14. - Weighted ANCOVA table for diabetes and MVPA.

(controlling for age, sex, body mass index, time of wearing the accelerometer, marital status, self-perceived health, self-perceived mental health, working status, season, smoking status (blood cotinine levels), education level and total household income).

	Estimate	SE	95% CI	t	*p*
Diabetes	-0.12	0.12	-0.34 – 0.11	-1	0.32
Age	-0.11	0.002	-0.01 – -0.007	-6.11	0.0002E-05
BMI	-0.34	0.005	-0.04 – -0.03	-7.17	0.0003E-08
Sex (women)	-0.27	0.04	-0.34 – -0.19	-7.06	0.0006E-08
Accelerometer wearing	0.1	0.01	0.07 – 0.12	8.3	0.0001E-11
Marital status (couple)	-0.17	0.06	-0.28 – -0.05	-2.89	0.004
Self-rated health	-0.41	0.07	-0.55 – -0.27	-5.64	0.0003E-04
Self-rated mental health	0.08	0.09	-0.08 – 0.25	0.98	0.33
Worked last year					
Study/retired	-0.23	0.13	-0.48 – 0.03	-1.73	0.08
Working	0.1	0.07	-0.03 – 0.23	1.49	0.14
Seasons					
Spring	-0.009	0.08	-0.16 – 0.14	-0.11	0.91
Summer	-0.05	0.06	-0.16 – 0.06	-0.92	0.36
Winter	-0.13	0.1	-0.33 – 0.08	-1.23	0.22
Levels of cotinine	-0.0002	0.0003E-01	-0.0003 – -0.0001	-5.93	0.0006E-05
Education					
High school	0.08	0.11	-0.14 – 0.3	0.68	0.5
Work school	0.07	0.1	-0.13 – 0.27	0.65	0.51
College	0.12	0.11	-0.1 – 0.33	1.07	0.29
University < Bachelor	0.19	0.1	0.002 – 0.38	1.98	0.05
Bachelor	0.32	0.12	0.07 – 0.56	2.55	0.01
University > Bachelor	0.31	0.11	0.09 – 0.53	2.8	0.005
Missing	0.07	0.1	-0.12 – 0.27	0.75	0.46
Household income					
$15k-$19,99k	-0.18	0.19	-0.56 – 0.2	-0.93	0.35
$20k-$29,99k	-0.25	0.15	-0.54 – 0.03	-1.74	0.08
$30k-$39,99k	-0.27	0.14	-0.54 – -0.007	-2.01	0.04
$40k-$49,99k	-0.19	0.14	-0.47 – 0.09	-1.31	0.19
$50k-$59,99k	-0.24	0.15	-0.55 – 0.06	-1.58	0.112
$60k-$79,99k	-0.11	0.14	-0.38 – 0.16	-0.78	0.44
$80k-$99,99k	-0.03	0.14	-0.3 – 0.25	-0.19	0.85
≥ $100k	0.05E-03	0.14	-0.27 – 0.27	0.01	1

Table S15. - Weighted ANCOVA table for diabetes and LPA.

(controlling for age, sex, body mass index, time of wearing the accelerometer, marital status, self-perceived health, self-perceived mental health, working status, season, smoking status (blood cotinine levels), education level and total household income).

	**Estimate**	**SE**	**95% CI**	**t**	***p***
Diabetes	-15.32	4.58	-24.3 – -6.34	-3.34	0.0009
Age	-0.78	0.14	-1.05 – -0.49	-5.33	0.0002E-03
BMI	-0.06	0.29	-0.63 – 0.5	-0.22	0.83
Sex (women)	-5.55	2.87	-11.18 – 0.08	-1.93	0.05
Accelerometer wearing	25.92	1.02	23.92 – 27.93	25.34	0.0002E-12
Marital status (couple)	7.57	3.45	0.08 – 14.34	2.19	0.03
Self-rated health	-18.34	4.09	-26.35 – -10.33	-4.49	0.0009E-02
Self-rated mental health	-4.14	5.19	-14.31 – 6.03	-0.8	0.43
Worked last year					
Study/retired	-6.58	5.19	-16.75 – 3.59	-1.27	0.21
Working	14.32	3.36	7.74 – 20.9	4.27	0.0002E-01
Seasons					
Spring	2.77	3.86	-4.79 – 10.33	0.72	0.47
Summer	3	5.51	-7.79 – 13.79	0.54	0.59
Winter	-13.43	4.75	-2.27 – -4.11	-2.82	0.005
Levels of cotinine	-0.005	0.003	-0.01 – 0.0005	-1.75	0.08
Education					
High school	24.71	10.57	3.98 – 45.43	2.34	0.02
Work school	5.21	10.72	-15.81 – 26.22	0.49	0.63
College	0.07	9.66	-18.86 – 19.01	0.008	0.99
University < Bachelor	-2.61	9.26	-20.75 – 15.54	-0.28	0.78
Bachelor	-10.05	10.31	-30.25 – 10.16	-0.98	0.33
University > Bachelor	-18.68	11.47	-41.15 – 3.79	-1.63	0.1
Missing	20.44	8.67	3.44 – 37.44	2.36	0.02
Household income					
$15k-$19,99k	0.26	12.13	-23.52 – 24.04	0.02	0.98
$20k-$29,99k	3.55	9.59	-15.24 – 22.34	0.37	0.71
$30k-$39,99k	7.93	8.38	-8.48 – 24.35	0.95	0.34
$40k-$49,99k	14.81	9.72	-4.25 – 33.86	1.52	0.13
$50k-$59,99k	12.7	8.34	-3.65 – 29.04	1.52	0.03
$60k-$79,99k	13.65	7.74	-1.51 – 28.82	1.77	0.08
$80k-$99,99k	15.87	8.31	-0.43 – 3.22	1.91	0.06
≥ $100k	36.96	7.12	-10.25 – 17.64	0.52	0.6

Table S16. - Weighted ANCOVA table for diabetes and steps.

(controlling for age, sex, body mass index, time of wearing the accelerometer, marital status, self-perceived health, self-perceived mental health, working status, season, smoking status (blood cotinine levels), education level and total household income).

	**Estimate**	**SE**	**95% CI**	**t**	***p***
Diabetes	-406.52	235.67	-868.42 – 55.37	-1.73	0.09
Age	-18.64	6.74	-31.84 – -5.44	-2.77	0.006
BMI	-82.56	14.62	-111.23 – -53.9	-5.65	0.0003E-04
Sex (women)	-884.22	143.06	-1164.62 – -603.81	-6.18	0.0001E-05
Accelerometer wearing	871.44	53.7	766.19 – 976.68	16.23	0.0002E-12
Marital status (couple)	-153.28	191.95	-529.49 – 222.94	-0.8	0.42
Self-rated health	-1002.82	209.55	-1413.54 – -592.1	-4.79	0.0002E-02
Self-rated mental health	-473.45	306.51	-1074.2 – 127.31	-1.55	0.12
Worked last year					
Study/retired	-101.03	294.05	-677.36 – 475.3	-0.34	0.73
Working	544.35	204.23	144.06 – 944.64	2.67	0.008
Seasons					
Spring	98.55	219.48	-331.62 – 528.73	0.45	0.65
Summer	128	201.73	-267.39 – 523.39	0.63	0.53
Winter	-794.22	218.69	-1222.84 – -365.59	-3.63	0.0003
Levels of cotinine	-0.5	0.14	-0.78 – -0.23	-3.6	0.0004
Education					
High school	712.38	375.73	-24.03 – 1494.04	1.9	0.06
Work school	416.55	381.11	-330.42 – 1201.55	1.09	0.27
College	63.03	358.14	-638.91 – 768.12	0.18	0.86
University < Bachelor	618.59	326.35	-21.04 – 1284.07	1.9	0.06
Bachelor	549.27	388.26	-211.71 – 1319.26	1.42	0.16
University > Bachelor	711.58	446.51	-163.57 – 1594.91	1.59	0.11
Missing	918.01	354.64	222.93 – 1641.82	2.59	0.01
Household income					
$15k-$19,99k	-612.01	479.64	-1552.09 – 328.07	-1.28	0.2
$20k-$29,99k	-357.9	452.46	-1244.69 – 528.9	-0.79	0.43
$30k-$39,99k	-260.05	393.61	-1031.51 – 511.41	-0.66	0.51
$40k-$49,99k	-247.87	493.48	-1215.06 – 719.33	-0.5	0.62
$50k-$59,99k	64.58	400.52	-720.43 – 849.59	0.16	0.87
$60k-$79,99k	-60.19	383.44	-811.72 – 691.35	-0.16	0.88
$80k-$99,99k	320.27	403.46	-470.5 – 1111.05	0.79	0.43
≥ $100k	209.96	350.32	-476.66 – 896.58	0.6	0.55

Table S17. - Weighted ANCOVA table for diabetes and SB.

(controlling for age, sex, body mass index, time of wearing the accelerometer, marital status, self-perceived health, self-perceived mental health, working status, season, smoking status (blood cotinine levels), education level and total household income).

	**Estimate**	**SE**	**95% CI**	**t**	***p***
Diabetes	12.78	6.25	0.53 – 25.03	2.05	0.04
Age	1.84	0.18	1.48 – 2.19	10.11	0.0002E-12
BMI	0.24	0.33	-0.41 – 0.88	0.73	0.47
Sex (women)	15.57	4.23	7.28 – 23.87	3.68	0.0003
Accelerometer wearing	34.85	1.2	32.5 – 37.2	29.02	0.0002E-12
Marital status (couple)	-1.48	3.37	-8.09 – 5.13	-0.44	0.66
Self-rated health	18.05	4.99	8.28 – 27.83	3.62	0.0003
Self-rated mental health	7	9.06	-10.77 – 24.76	0.77	0.44
Worked last year					
Study/retired	-6.69	6.04	-18.54 – 5.15	-1.11	0.27
Working	-23.12	5.17	-33.25 – -12.99	-4.47	0.0001E-01
Seasons					
Spring	-1.98	4.8	-11.38 – 7.43	-0.41	0.68
Summer	-13.38	6.29	-25.71 – -1.06	-2.13	0.03
Winter	13.47	5.85	2 – 24.94	2.3	0.02
Levels of cotinine	-0.0007	0.004	-0.008 – 0.007	-0.17	0.87
Education					
High school	-5.08	10.37	-25.41 – 15.25	-0.64	0.62
Work school	14.59	10.6	-6.19 – 35.37	1.27	0.17
College	14.7	11.54	-7.91 – 37.31	-0.49	0.2
University < Bachelor	24.82	9.56	6.09 – 43.56	2.6	0.01
Bachelor	23.18	9.55	4.47 – 41.9	2.43	0.02
University > Bachelor	19.52	15.03	-9.94 – 48.97	1.3	0.19
Missing	-5.16	8.06	-20.95 – 10.64	-0.64	0.52
Household income					
$15k-$19,99k	9.84	13.77	-17.14 – 36.83	0.72	0.48
$20k-$29,99k	8.46	13.17	-17.36 – 34.28	0.64	0.52
$30k-$39,99k	0.78	12.8	-24.3 – 25.87	0.06	0.95
$40k-$49,99k	-2.43	12.92	-27.76 – 22.9	-0.19	0.85
$50k-$59,99k	5.43	12.33	-18.75 – 29.59	0.44	0.66
$60k-$79,99k	-6.75	12.44	-31.13 – 17.63	-0.54	0.59
$80k-$99,99k	-2.95	13.04	-28.5 – 22.61	-0.23	0.82
≥ $100k	5.94	11.57	-16.73 – 28.61	0.51	0.61

© 2019 by the authors. Submitted for possible open access publication under the terms and conditions of the Creative Commons Attribution (CC BY) license (http://creativecommons.org/licenses/by/4.0/).
